# From John Snow to omics: the long journey of environmental epidemiology

**DOI:** 10.1007/s10654-018-0398-4

**Published:** 2018-04-21

**Authors:** Paolo Vineis

**Affiliations:** 1 MRC-PHE Centre for Environment and Health, Imperial College London, Norfolk Place, London, W21PG UK; 2 Italian Institute for Genomic Medicine, Turin, Italy

**Keywords:** Non-communicable disease, Multicausality, Omics, Exposomics, Epigenetics, Socially-transmitted conditions

## Abstract

A major difference between infectious and non-communicable diseases is that infectious diseases typically have unique necessary causes whereas noncommunicable diseases have multiple causes which by themselves are usually neither necessary nor sufficient. Epidemiology seems to have reached a limit in disentangling the role of single components in causal complexes, particularly at low doses. To overcome limitations the discipline can take advantage of technical developments including the science of the exposome. By referring to the interpretation of the exposome as put forward in the work of Wild and Rappaport, I show examples of how the science of multi-causality can build upon the developments of omic technologies. Finally, I broaden the picture by advocating a more holistic approach to causality that also encompasses social sciences and the concept of embodiment. To tackle NCDs effectively on one side we can invest in various omic approaches, to identify new external causes of non-communicable diseases (that we can use to develop preventive strategies), and the corresponding mechanistic pathways. On the other side, we need to focus on the social and societal determinants which are suggested to be the root causes of many non-communicable diseases.

Among the heroes of modern epidemiology, John Snow is probably the most popular from the viewpoint of both epidemiologists and the lay public. By removing the handle of the Broad Street Pump in London, Snow allegedly stopped the epidemic of cholera (though the story is slightly more complex; for a better account see [1]). This public health gesture was supported by parallel work on mapped results that indicated that distributions of the diseased followed the patterns of water provision from water companies (http://www.ph.ucla.edu/epi/snow/snowbook3.html). Another hero in epidemiology, Max von Pettenkofer, initiated a public health revolution by improving sanitation and housing in Munich. Pettenkofer was an anti-contagionist, i.e. he did not believe that the then predominant infectious diseases could be attributed to germs. Though anti-contagionists were wrong, they have attracted much attention and favour because of the effectiveness of their preventive practices, which were focused—to use a now popular expression—on the “causes of causes”. It is relevant to postulate whether these and other early movements for healthy cities in the nineteenth century can be replicated in reaction to the current epidemics of so-called “non-communicable diseases” (NCDs). Thus, in this commentary the analogies between concepts from the period of Snow and Pettenkofer and today’s challenges posed by understanding NCDs spreading in the world are discussed.

The reasoning behind this paper can be summarized as follows: a major difference between infectious and non-communicable diseases is that infectious diseases typically have unique necessary causes whereas non-communicable diseases typically have many causes which by themselves are usually neither necessary nor sufficient. Epidemiology seems to have reached a limit in disentangling the role of single components in causal complexes, and to overcome limitations the discipline can take advantage of technical developments including the science of the exposome. By referring to the interpretation of the exposome as put forward in the work of Wild and Rappaport, I show examples of how the science of multi-causality can build upon the developments of omic technologies. Finally, I broaden the picture by advocating a more holistic approach to causality that also encompasses social sciences and the concept of embodiment.

## Multicausality: real or due to lack of knowledge?

By simplifying Snow’s story, we can say that the “*Snow manoeuvre*” (the closure of the pump) was successful for a number of reasons that do not apply to NCDs: *Vibrio* (not known at the time if we exclude the work of Filippo Pacini) was a unique and necessary cause of cholera, i.e. the interruption of the causal chain was allowed because of a single act. However, if we consider the current leading diseases in the world according to the Burden of Disease programme, they are not due to necessary causes. For example, hypertension arises as a consequence of a network of determinants including excessive salt in one’s diet. The well-known characteristics of NCDs have led to the basic concept of NCD epidemiology, multifactoriality as epitomized by “Rothman’s pies” [2]. Causation is interpreted as a chain of events or a constellation of exposures where none by itself is able to cause the disease. This model has far reaching consequences for the theories of causality. In the case of cancer, for example, it is hypothesized that several mutations, epimutations or “hallmarks of cancer” [3] are needed to complete a causal chain from exposure to disease, though the exact number of events is unknown. This implies that in any population there are some individuals who may be especially predisposed to developing cancer because of inherited genetic variations, mutations induced by carcinogenic substances, epigenetic changes, and/or other “hallmarks of cancer”. A small number of people in the population may lack the activation of just the last stage or hit to complete the carcinogenic process—even at low exposure doses, while a larger number may lack the activation of two stages, three stages, etc. Luckier individuals (but often based on their lower exposures to risk factors) have no activated stage. With exceptions (HPV and cervical cancer being the most obvious one), we do not know single necessary causes for any NCD. Then the question arises: is this a consequence of our ignorance or is it a fact?

Ignorance, as an explanation of the web of causation, is similar to the unawareness that existed before the discoveries of microbiology when diseases were classified based on the symptoms, while with technological developments (microscope, laboratory glassware, enriched cultures) the discovery of single infectious agents led to a dramatic reclassification (e.g. some “fevers” became TB and others malaria). Therefore, according to the “ignorance” thesis we might be on the edge of a new revolution in our knowledge of NCDs, should a technological development allow us to isolate single necessary causes for well-identified, redefined disease entities.

This seems unlikely to me. The last 50 years of epidemiology have seen the greatest effort in history to identify the causes of cancer and other NCDs, with many successes (mainly coming from epidemiology) and also much frustration. It seems that we are now accepting the multicausality paradigm, by which there are far reaching implications. For example, when the International Agency for Research on Cancer (IARC) Working Groups concluded that secondhand smoke, processed red meat and ambient air pollution are carcinogenic to humans, they also noted that for the dose–response relationship no threshold was evident. This in turn implies that all three agents are likely to act synergistically with other exposures, i.e. neither agent is a single necessary cause of cancer. In brief, we accept that something is carcinogenic to humans when it can act at low doses and is not necessary nor sufficient to induce the disease. We also know from extensive research that each of the three exposures can cause other NCDs: for example, meat intake is associated with cardiovascular diseases and air pollution with cardiovascular, respiratory and perhaps neurological diseases. This line of reasoning seems to be the only one supported by current evidence but is not accepted by people with vested interests, including toxicologists sponsored by industries, who seem to wait for the next revolution in causality that will put things in order with NCDs.

In practice, if we accept that there is no alternative to the multicausality model for NCDs, we need to develop tools that will allow us to investigate networks and pathways to lend credibility to causal chains, to allow the detection of early changes at low dose levels, and to study synergies between exposures and components of mixtures. Also, we need to understand how early exposures can leave marks that may impact health outcomes after decades, like in the case of the Dutch famine and its impact later on in life (see below). I believe that the concept of the exposome and the associated technologies provide such tools, as exemplified in the Exposomics project (http://www.exposomicsproject.eu). In support of this, I discuss a few examples where the exposome can be used to inform causality, and particularly for NCDs. What follows does not mean to be an exhaustive description of what the exposome science is or might be, but rather provide some examples of contributions in key aspects of the multi-causality conundrum.

## The science of multicausality: early findings from Exposomics

### Omics indicate early molecular changes at low levels of exposure

The epidemiology of NCDs has been struggling with how to determine effects of exposures at low doses. There is no evidence that common exposures such as air pollution or secondhand smoking show a threshold in their action. For example, in the ESCAPE network we found that mortality increases below the current thresholds set by WHO [4]. The effects of low or very low doses may be due to genetic susceptibility, as researchers have argued for years, but in fact genetic epidemiology so far has been unable to find sets of gene variants that are strongly associated with increased susceptibility (except for familial conditions such as BRCA1 mutations). Even in the case of smoking and lung cancer, the additional susceptibility conferred by gene variants tends to be modest [5]. Another explanation of low dose effects, as suggested above, is the combination of different exposures conferring acquired susceptibility.

**Fig. 1:**
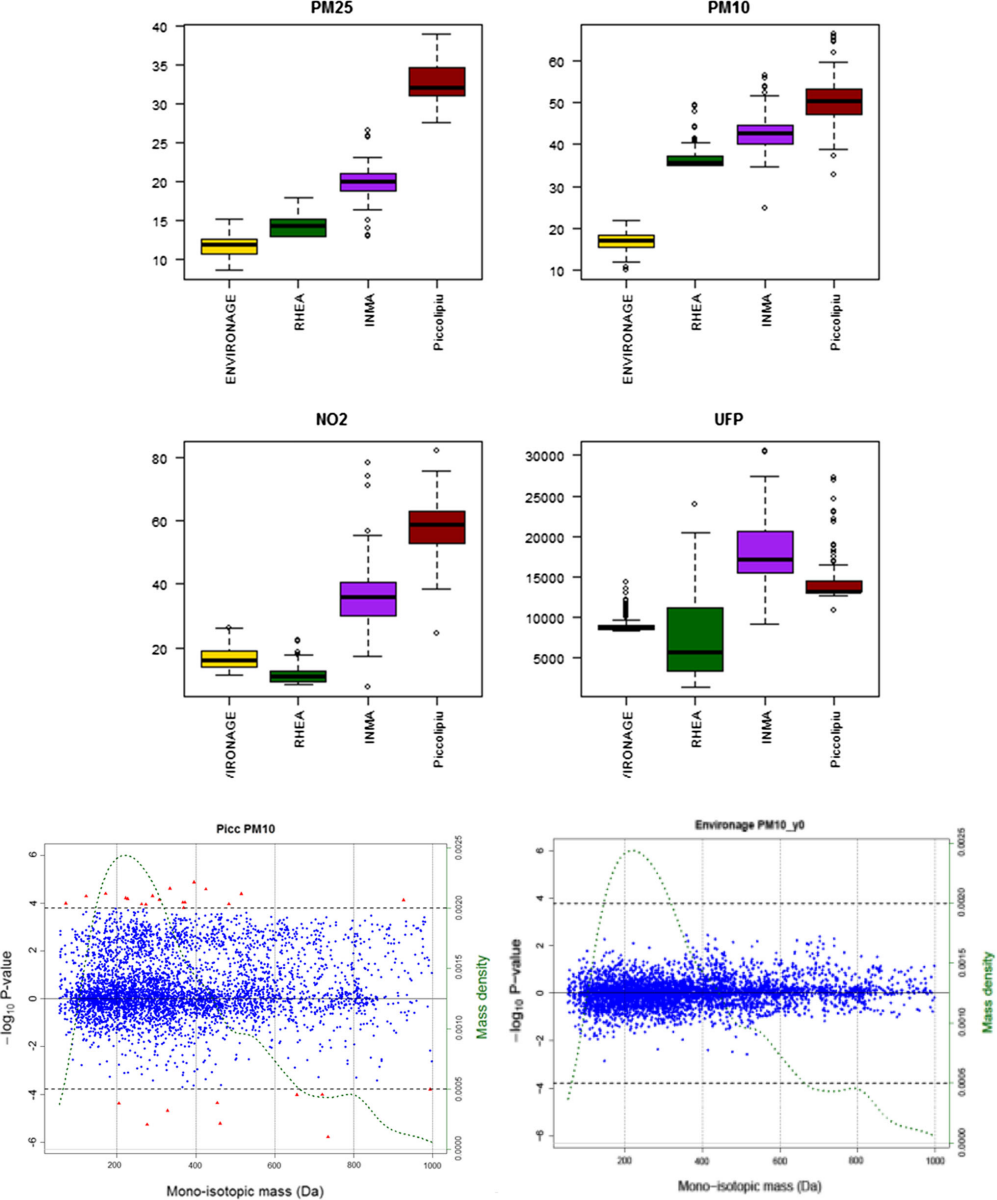
PM_10_ by cohort (top) and metabolomic signals (bottom) in Piccoli + (higher exposure levels) and in Environage (low exposure levels). In red metabolomic signals below the Bonferroni threshold of *p* = 1.63 × 10^−4^. UFP = ultrafine particles (from Exposomics, unpublished)

The existence of low dose effects implies that we should be able to detect molecular changes at those levels of exposure. Support for this is starting to appear through the application of ‘omics in investigations of low dose exposures. For example, we have found that the levels of exposure to PM_10_ experienced in utero from four different European areas (Fig. 1a) influence cord blood metabolomic signals (Fig. 1b). This can be perceived at low levels of exposure to air pollution, though we also note that the effects are stronger in the areas with higher levels of pollution than in rural areas with lower levels (unpublished).

### Omics suggest that different pollutants in a mixture may lead to different biological pathways, but perhaps not always

**Fig. 2:**
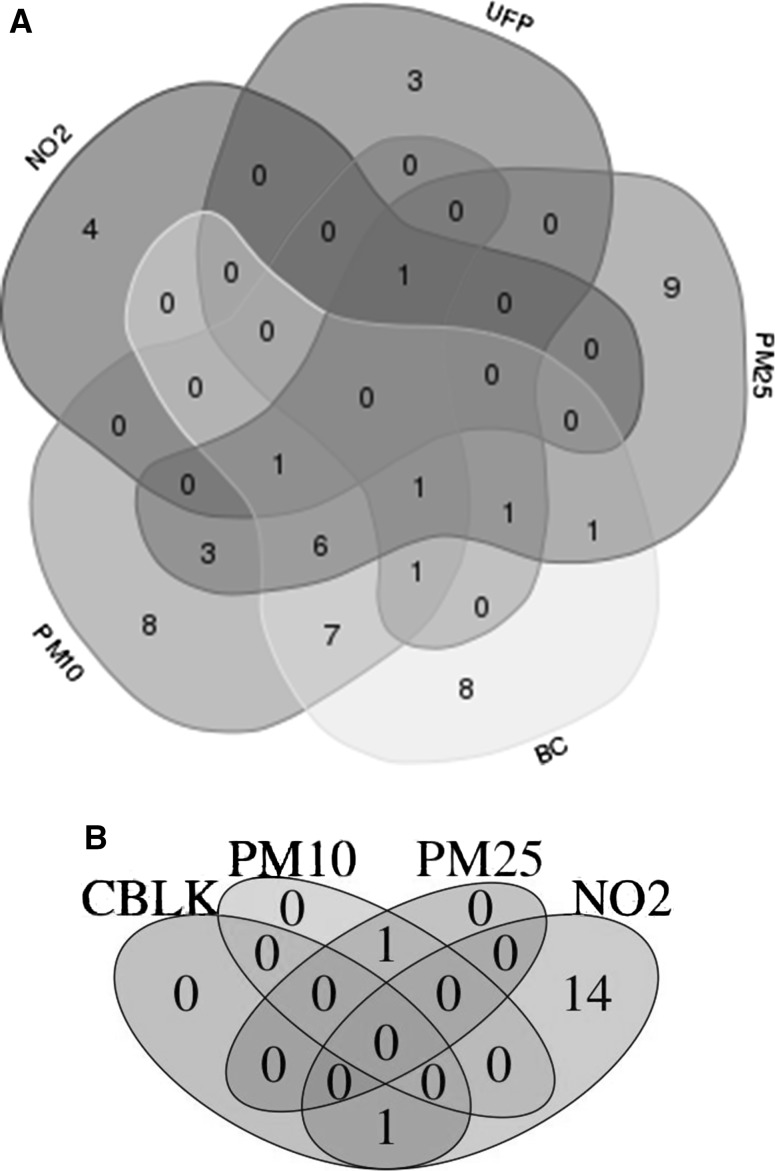
Pollutant-specific miRNAs (**a**) or metabolomic changes (**b**) associated with traffic-related air pollution (TRAP) exposure. **a** The figure shows the overlap as well as the specificity of the pollutant-specific circulating miRNAs associated with exposure to NO2, UFP, PM2.5, BC and PM10 of subjects in Hyde Park and Oxford Street. Krauskopf et al. [23]. **b** Metabolomic signatures of different components of air pollution (Oxford Street study) (Bonferroni significance) (Van Veldhoven et al. unpublished)

**Fig. 3:**
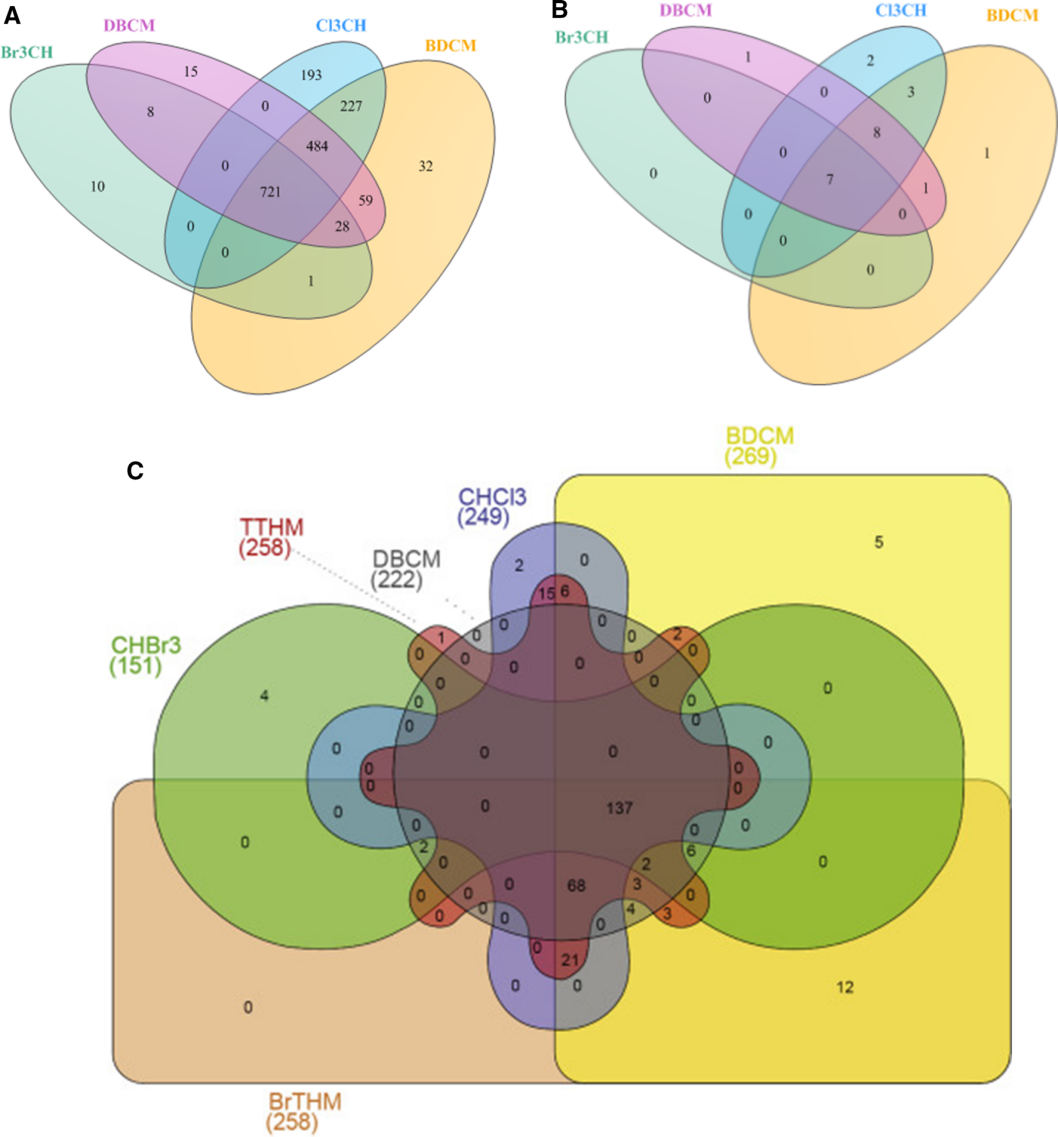
In the Piscina study transcriptomic signals (**a**), miRNAs (**b**) and metabolomic signals (**c**) overlap across different disinfection by-products (DBP) in a swimming pool. Venn diagrams show Bonferroni significant hits, adjusted for sex, age and BMI. CHCl_3_ = chloroform; BDCM = bromodichloromethane; DBCM = dibromochloromethane; Br_3_CH = bromoform (N = 41) (Espin et al.[24] and Van Veldhoven et al. [7])

Do components in a mixture act separately (via different metabolic or molecular pathways) or do they impact common pathways? Omics investigations shown in Figs. 2 and 3 suggest that both cases are realistic. Figure 2 refers to the Oxford Street randomized cross-over trial [6] in which volunteers were exposed to high (Oxford Street, London) or low (Hyde Park) levels of air pollution. The figure shows that both for RNA (gene expression and circulating miRNA, involved in gene expression modulation) and for metabolites from mass spectrometry the different components of air pollution give rise to signals that do not overlap, suggesting that each pollutant (except perhaps PM2.5 and PM10) follows a different metabolic or molecular pathway to exert its effects. Again, these are low or very low levels of exposure. There are limitations in our ability to identify clear pathways, particularly in metabolomics due to still poor annotation of signals, but Fig. 2 seems to open an interesting avenue for research.

The example shown in Fig. 3 is opposite. In this case we enrolled swimmers (volunteers) to swim in a normal Barcelona pool contaminated by chlorinated or brominated disinfection-by-products [7]. In this example there seems to be a broad overlap between miRNA and, respectively, MS metabolomic signals across five different disinfection by-products.

### Omics suggest that meaningful biological pathways connect exposure and outcome

The IARC Monographs use a strict procedural approach to the evaluation of causality, as explained in their Preamble [8]. They use criteria very similar to the Bradford Hill guidelines used by epidemiologists for decades and derived from Henle–Koch’s postulates for infectious diseases. However, an extension of Henle–Koch’s postulates to NCDs is not straightforward, since they stated that “1. The agent must be demonstrable in every case of the disease; 2. The agent is not present in other diseases; 3. After isolation in culture, the agent must be able to produce the disease in experimental animals”. Clearly this is at odds with what we have said about NCDs, and the postulates have been modified by Bradford Hill to be adapted to the multifactorial nature of NCDs. Among Bradford Hill’s guidelines there is also reproducibility in animals and biological plausibility, two criteria extensively applied in the IARC Monographs to establish causality. For example, red meat intake has been associated with exposure to at least four different groups of carcinogenic substances [9, 10].

**Fig. 4:**
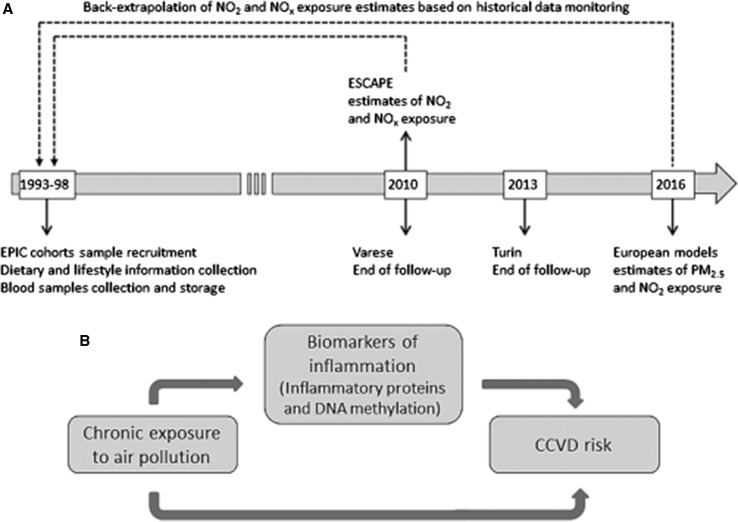
Oxidative stress and inflammation mediate the effect of air pollution on cardio‐ and cerebrovascular disease: A prospective study in nonsmokers. Fiorito et al. [11]

One way to lend credibility to a causal interpretation of epidemiological associations is to look for intermediate steps that link exposure and disease, an approach we have called “meet-in-the-middle”. In the case of air pollution, many studies (and in particular the ESCAPE network) have demonstrated an impact on cardiovascular diseases (CVD), and several studies also an impact of air pollution on inflammatory and oxidative stress markers, but none has linked the three components together, i.e. exposure, intermediate mechanisms and outcome. In Exposomics we designed a case–control study on CVD nested in the EPIC cohort. We measured air pollution, inflammatory biomarkers, and whole-genome DNA methylation in blood collected up to 17 years before the diagnosis. We identified enrichment of altered DNA methylation in ‘ROS/Glutathione/Cytotoxic granules’ and ‘Cytokine signaling’ pathways related genes, associated with both air pollution and with CVD risk [11]. Our findings indicate that chronic exposure to air pollution can cause oxidative stress, which in turn activates a cascade of inflammatory responses mainly involving the ‘Cytokine signaling’ pathway, leading to increased risk of CVD. Inflammatory proteins and DNA methylation alterations can be detected several years before CVD diagnosis in blood samples, being promising pre-clinical biomarkers. Figure 4 summarizes our “meet-in-the-middle” reasoning.

### Epigenetics suggests that stem cells may have long term memory of environmental challenges

Exposures in early life may leave a long-lasting trace in the body, with effects that can manifest after many decades. This concept was at the basis of Barker’s hypothesis [12] and of the current developmental origin of health and disease (DOHaD) theory [13]. After 60 years after being exposed to the Dutch famine in utero individuals showed epigenetic changes in genes including IGF-2 [14] (though the evidence is far from conclusive). These observations have been made from circulating white blood cell DNA, and the only way to make sense of them is that epigenetic changes are transmitted through generations of cells via stem cells. We know that some exposures alter the structure of DNA causing mutations, but there is increasing evidence that the same or other environmental exposures, including those that occur during foetal development in utero, can cause epigenetic effects that modulate gene expression without altering DNA structure. Some of such epigenetic changes are at least partially reversible, but other epigenetic modifications seem to persist even for decades. In addition to the Dutch famine, probably the best example is tobacco smoking. In a series of epigenome-wide association studies we have investigated the dynamics of smoking-induced epigenetic changes after smoking cessation. Two distinct classes of CpG sites were identified: sites whose methylation reverted to levels typical of never smokers within decades after smoking cessation, and sites remaining differentially methylated, even more than 35 years after smoking cessation [15]. This and other similar studies highlight persistent epigenetic markers of smoking, which can potentially be detected decades after cessation.

To explain the long-term persistence of epigenetic modifications, such as DNA methylation, we proposed an analogy with immune memory. We proposed that an epigenetic memory can be established and maintained in self-renewing stem cell compartments. We suggested that the observations concerning early life effects on adult diseases (the Dutch famine) and the persistence of methylation changes in ex-smokers support our hypothesis. Although epigenetic changes seem to be mainly adaptive, they are also likely implicated in the pathogenesis and onset of diseases. Elucidating the relationships between the adaptive and maladaptive consequences of the epigenetic modifications that result from complex environmental exposures is a major challenge for current and future research in epidemiology and epigenetics [16].

Like the methods of Snow and Pettenkofer today’s epidemiologist uses patterns to suggest potential cause and effect relationships, before clear mechanisms underlying associations between exposures and outcomes are defined. However, unlike at their time, as we have argued here, we have a new toolbox to expand our methodologies and to better support causality with potential mechanisms, particular for NCDs. However, there are still considerable underlying challenges that we face in understanding the full picture of causality for NCDs, as I try to argue in the next paragraph.

## The most difficult challenge of causality and NCDs: to connect natural with social sciences

What are NCDs? A recent debate in Lancet Global Health, sparked by Allen and Feigl, focused on the ambiguous and unclear nature of NCDs [17]. According to the authors, “The current list of NCDs describes a ragtag group of leftovers that do not satisfy Koch’s postulates”. The attributes of this mixed bag are, among others: chronicity; global burden; preventable nature; common proximal risk factors (cholesterol, blood pressure, glucose, obesity); common behavioural risk factors (smoking, alcohol, diet, inactivity, etc.); common distal risk factors (economic, social, environmental); common issues of inequality and injustice. Given these shared properties and the ambiguous nature of their current denomination (NCDs), Allen and Feigl suggest we call them “Socially-Transmitted Conditions” (STC). This is an interesting (and controversial) move, but leads to an even more difficult challenge, i.e. how we connect the “social” and the “natural”, the study of society and the study of bodies and molecules to investigate the causes of NCDs. This problem has been named by Nancy Krieger the *embodiment* of social relationships [18]. Once again, we suggest that the concepts and tools of exposomics can be instrumental in accomplishing this goal. One example of how omics (namely, epigenomics) can connect social determinants of health with molecular changes is in the association between “age acceleration” and socio-economic status.

Low socioeconomic position (SEP) has been associated with earlier onset of age-related chronic conditions and reduced life-expectancy. We have investigated the association of SEP with DNA methylation age acceleration (AA) in more than 5000 individuals belonging to three independent prospective cohorts from Italy, Australia, and Ireland [19]. AA is based on a discrepancy between chronological age and the level of methylation of a number of CpG islands in DNA, a consistent indicator of biological ageing. Low SEP was associated with greater AA and the association was only partially modulated by the unhealthy lifestyle habits of individuals with lower SEP. Individuals who experienced life-course SEP improvement had intermediate AA compared to extreme SEP categories, supporting the relative importance of early childhood social environment [19].

This example is only one among other examples of *embodiment*, and a suite of indicators has been proposed and used in our Lifepath network on SEP-related ageing trajectories (www.lifepathproject.eu), e.g. allostatic load, inflammatory biomarkers, metabolomics, proteomics [20] and transcriptomics [21]. Also, the conceptual understanding of the complex relationships between SEP (an overarching determinant), risk factors for NCDs, molecular and metabolic mechanisms and health outcomes is far from being understood and cannot be tackled with simplistic or reductionist explanations [22].

## Conclusions

There are two main messages in what precedes. First, something equivalent to the “Snow manoeuvre” is unlikely to be realistic for NCDs. NCDs are different from infectious diseases indeed. Infectious diseases, in general (not always), are due to necessary agents that are very specific (e.g. Mycobacterium for TB, Vibrio for cholera, HIV for AIDS, etc.). Specificity is such that also medical preventive measures (i.e. vaccines) are disease-specific. Rather than single causes with short induction periods in NCDs we are looking for complex webs of causation, in which multiple factors—including at low doses—are involved; and *embodiment* of social relationships and social structure is likely to be a key concept. To respond to these challenges the traditional tools of epidemiology are inadequate. Like the substantial progress in the identification of causes and the classification of infectious diseases were propelled by the development of new tools, including the microscope, now the new technologies (largely molecular biology and mass spectrometry) may allow important steps forward in NCD epidemiology. However, we have to be aware that technological advancements are never sufficient in the absence of a clear conceptual framework to interpret them. Two of such frameworks (obviously mutually compatible) are the theory of “embodiment” [18] and the concept of socially-transmitted conditions [17].

In fact, and this is the second point I want to make, anti-contagionists were right in saying (and demonstrating) that “systemic” societal interventions in improving the health of cities had multiple benefits: sanitation and fresh water led to a decline of a number of water-borne diseases, not just one or a few. Improvements of air and housing quality were also accompanied by similar broad, wide-ranging benefits. This is clearly still true for NCDs (or socially-transmitted conditions), as they tend to share the same, or at least part of the same risk factors. This concept is not only true for physical health but also for mental health. Despite vast research in the area, we know little of the etiologies underlying mental health (e.g. depression, the leading cause of disability in high-income countries), however it is likely that similar improvements and systemic interventions would improve mental health on a societal level. As housing density and sanitation have led to successes in Pettenkofer’s Munich, nowadays city planning and the organization of life including leisure time may have much to do with the development of NCDs and adverse mental health.

In conclusion, it is likely that to tackle NCDs effectively on one side we need to invest in various omic approaches, to identify new external causes of non-communicable diseases that we can use to develop preventive strategies. On the other side, we need to focus much more on the social and societal determinants which are suggested to be the root causes of many non-communicable diseases.
